# A Novel 14mer Peptide Inhibits Autophagic Flux via Selective Activation of the mTORC1 Signalling Pathway: Implications for Alzheimer’s Disease

**DOI:** 10.3390/ijms252312837

**Published:** 2024-11-29

**Authors:** Cloe García Porta, Kashif Mahfooz, Joanna Komorowska, Sara Garcia-Rates, Susan Greenfield

**Affiliations:** Neuro-Bio Ltd., Building F5, Culham Science Centre, Abingdon OX14 3DB, UK

**Keywords:** mTORC1, T14, NBP14, rapamycin, Alzheimer’s disease, autophagy

## Abstract

During development, a 14mer peptide, T14, modulates cell growth via the α-7 nicotinic acetylcholine receptor (α7 nAChR). However, this process could become excitotoxic in the context of the adult brain, leading to pathologies such as Alzheimer’s disease (AD). Recent work shows that T14 acts selectively via the mammalian target of rapamycin complex 1 (mTORC1). This pathway is essential for normal development but is overactive in AD. The triggering of mTORC1 has also been associated with the suppression of autophagy, commonly observed in ageing and neurodegeneration. We therefore investigated the relationship between T14 and autophagic flux in tissue cultures, mouse brain slices, and human Alzheimer’s disease hippocampus. Here, we demonstrate that T14 and p-mTOR s2448 expression significantly increases in AD human hippocampus, which was associated with the gradual decrease in the autophagosome number across Braak stages. During development, the reduction in T14 positively correlated with pTau (Ser202, Thr205) and two selective autophagy receptors: p62 and optineurin. In vitro studies also indicated that T14 increases p-mTOR s2448 expression, resulting in the aggregation of polyubiquinated substances. The effective blockade of T14 via its cyclic variant, NBP14, has been validated in vitro, in vivo, and ex vivo. In this study, NBP14 significantly attenuated p-mTOR s2448 expression and restored normal autophagic flux, as seen with rapamycin. We conclude that T14 acts at the α-7 receptor to selectively activate the mTORC1 pathway and consequently inhibit autophagic flux. Hence, this study describes a further step in the process by which T14 could drive neurodegeneration.

## 1. Introduction

Autophagy is a process by which a cell breaks down old or abnormal proteins in its cytoplasm. As such, it plays an essential function in the nervous system, enhancing synaptic pruning during development as well as in ageing and neurodegeneration [1]. Recent studies have established that decreased autophagic activity is associated with Alzheimer’s disease (AD) pathogenesis [2]. Additionally, the accumulation of anomalous Tau and amyloid β1–42 (Aβ1–42) has been linked to lysosomal dysfunction in AD [3]. Patients’ brains have shown a build-up of immature autophagic vacuoles (AVs) in dystrophic neurites, indicating a disturbance in autophagy [4].

An important factor driving AD neurodegeneration could be the 14mer peptide, T14 [5], cleaved from the C-terminus of acetylcholinesterase (AChE) [6]. This enzyme possesses numerous trypsin-like cleavage sites, permitting the synthesis of T30, a 30mer peptide with bioactive properties analogous to those of T14, which it encompasses, as well as T15, a 15mer inert peptide [5,7]. T14 has a trophic function in early development, where it regulates calcium influx and, in turn, cell growth via the α-7 nicotinic acetylcholine receptor (α-7 nAChR). However, its aberrant reactivation during adulthood has been postulated to drive AD [8,9]. Here, it stimulates the excitotoxic influx of calcium into cells, initiating glycogen synthase kinase-3 (GSK-3) activity [10,11], followed by the triggering of mammalian target of rapamycin complex 1 (mTORC1) [12,13], and then the release of AChE from the dendritic smooth endoplasmic reticulum (SER) and other intracellular storage [7]. The newly released T14 disperses into the extra-synaptic space to act on α7 nAChR, perpetuating the noxious effects in adjacent cells [14]. mTORC1 also initiates Tau phosphorylation (p-Tau) [15] and amyloid beta (Aβ) [5,16] cleavage from amyloid-beta precursor protein (APP), which contributes to T14 toxicity and leads to neurodegeneration and drives metastasis [8,17,18,19]. Our previous work has established that T14 selectively activates the mTORC1 pathway, which is vital for its downstream intracellular effects [10]. The cyclic version of the peptide, NBP14, effectively inhibits T14 by displacing its endogenous linear counterpart [9,18].

This study investigated two of the mechanisms involved in the activation of autophagy: the Atg8 and the mTOR systems. mTOR is a known regulator of metabolism and growth [20], which also plays a role in monitoring autophagic flux. The hyperactivation of mTORC1 has been proposed to drive AD [21,22] but also inhibit the catabolic stages of autophagy [4]. In the Atg8 pathway, the lipidation of the microtubule-associated protein 1 light chain 3 beta (LC3B) isoform I into LC3B phosphatidylethanolamine (LC3B-II) is crucial for autophagosome formation [23,24]. Once this structure reaches maturation, lysosomal fusion generates autolysosomes, mediated by SNARE (soluble N-ethylmaleimide-sensitive factor attachment protein receptor) [25]. During the final stage, lysosomal enzymes degrade the inner membrane and its cargo. However, the resulting macromolecules, such as fatty acids, amino acids, or sugars, are transported inside lysosomes for recycling [26]. The autophagosome turnover rate can be measured via selective autophagy receptors, such as Sequestome 1 (SQSTM1/p62) and optineurin, that bind polyubiquitinated proteins to join the complete autophagosome via the selective targeting of the LC3-interacting region (LIR) motif [27].

Given the association between autophagy and AD and the role of T14 in development, ageing, and pathogenesis, we investigated whether this peptide influences autophagic flux in human brain tissue from Alzheimer’s disease patients with varying degrees of severity (Braak staging), mouse brains, and PC12 cells. We then determined whether the selective blockade of mTORC1 via NBP14 reverses the T30-induced effects in the autophagy of PC12 cells to the same degree as rapamycin.

## 2. Results

### 2.1. The Increase in T14 Across AD Braak Stages Is Linked to Decreased Autophagy

**Figure 1:**
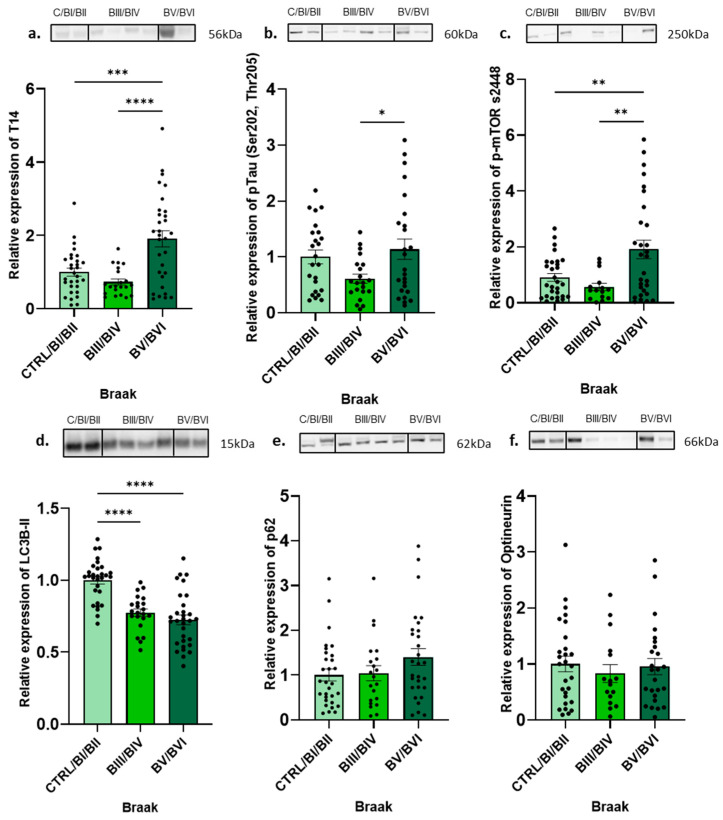
Quantification of T14, pTau, mTORC1, and autophagic markers across Braak stages. (**a**) Expression of T14 (37 kDa) compared to Control/Braak stage I/II (CTRL/BI/BII). (**b**) Expression of pTau (Ser202, Thr205) (60 kDa) compared to CTRL/BI/BII. (**c**) Expression of p-mTOR s2448 (250 kDa) compared to CTRL/BI/BII. (**d**) Expression of LC3B-II (15 kDa) compared to CTRL/BI/BII. (**e**) Expression of p62 (62 kDa) compared to CTRL/BI/BII. (**f**) Expression of optineurin (66 kDa) compared to CTRL/BI/BII. Representative Western blots are shown above each graph; all data were normalised relative to total protein via Ponceau stain. The bars represent the mean, the errors represent the SEM, and the dots represent individual data points. One-way ANOVA with post hoc Tukey’s tests, * *p* < 0.05, ** *p* < 0.01, *** *p* < 0.001, **** *p* < 0.0001, and *n* = 22–31/Braak stage.

We initially aimed to investigate whether the increase in T14 associated with the development of AD was also linked to compromised autophagic flux. A total of 83 postmortem human hippocampus samples were analysed across three Braak stages: CTRL/BI/BII, Braak stage III/IV (BIII/BIV), and Braak stage V/VI (BV/BVI). The T14 values were seen to significantly increase in the last Braak stages (Figure 1a, post hoc Tukey’s, F = 14.33, *p* < 0.0001, R^2^ = 0.2662). The same trend was observed with pTau (Ser202, Thr205) (Figure 1b, post hoc Tukey’s, F = 3.573, *p* < 0.05, R^2^ = 0.096) and p-mTOR s2448 expression (Figure 1c, post hoc Tukey’s, F = 7.452, *p* < 0.002, R^2^ = 0.1755). The autophagosome number, represented by LC3B-II, was shown to gradually decrease as AD advanced (Figure 1d, post hoc Tukey’s, F = 25.74, *p* < 0.0001, R^2^ = 0.3916), displaying a significant difference between the CTRL/BI/BII, BIII/IV, and BV/VI, but not between those last two stages. No significant differences were noticed when staining with the autophagy markers. Nonetheless, Braak stage BV/BVI had the highest amount of p62 (Figure 1e, post hoc Tukey’s, F = 1.919, *p* < 0.2, R^2^ = 0.04728) and optineurin aggregation compared to that in the early Braak stages (Figure 1f, post hoc Tukey’s, F = 0.2951, *p* < 0.8, R^2^ = 0.008863).

### 2.2. Under Healthy Conditions, Autophagic Flux Increases During Mouse Development

**Figure 2:**
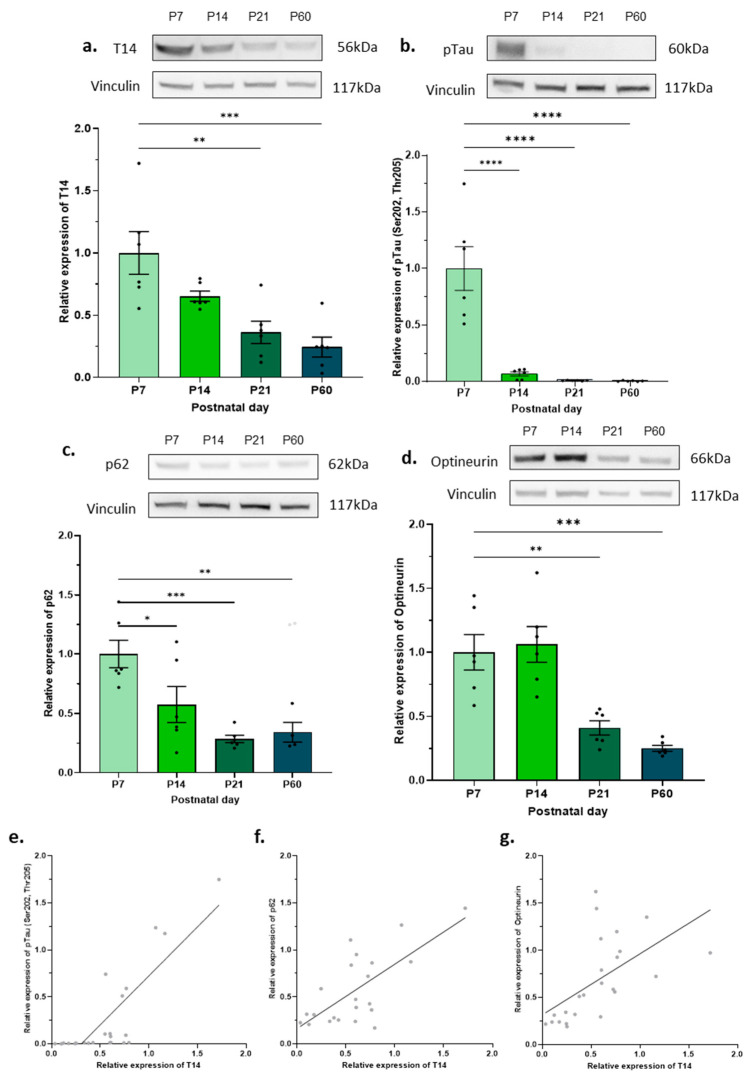
Measurement of T14, pTau, and autophagy markers in developing murine brain. (**a**) Expression of T14 (56 kDa) during development relative to the mean of P7. (**b**) Expression of pTau (60 kDa) during development relative to the mean of P7. (**c**) Expression of p62 (62 kDa) during development relative to the mean of P7. (**d**) Expression of optineurin (66 kDa) during development relative to the mean of P7. Representative Western blots for each protein and their corresponding vinculin (117 kDa) measurements are shown above each graph. All data were normalised to vinculin. The bars represent the mean, the errors represent the SEM, and the dots represent the individual data points of P7, P14, P21, and P60 mice. One-way ANOVA with post hoc Dunnett’s tests, * *p* < 0.05, ** *p* < 0.01, *** *p* < 0.001, **** *p* < 0.0001, *n* = 6/age. Spearman’s rank correlations between T14 and (**e**) pTau, (**f**) p62 and (**g**) optineurin (*n* = 6/group, except for *n* = 4 P60 mice in p62 data, as in (**c**)). Dots represent individual mice; line represents simple linear regression.

Once the relationship between T14 and autophagy was explored in AD pathology, we wanted to determine whether that association is also present during development. T14 expression significantly decreased with age, with the P7 mice having the most and the P60 mice having the least T14 (Figure 2a, post hoc Dunnett’s, F _(3,20)_ = 10.01, *p* < 0.0005, R^2^ = 0.6002). The same trend was seen with pTau (Ser202, Thr205), where its expression also declined with age (Figure 2b, post hoc Dunnett’s, F _(3,20)_ = 25.17, *p* < 0.0001, R^2^ = 0.7906). The expression of the autophagy marker p62 gradually decreased with age, with the P7 mice showing the highest levels (Figure 2c, post hoc Dunnett’s, F _(3,18)_ = 9.016, *p* < 0.0008, R^2^ = 0.6004). Correspondingly, the optineurin expression also declined with age. Although the P7 optineurin readings were not significantly higher than those from the P14 mice, they were significantly higher than in the P21 and P60 mice (Figure 2d, post hoc, F _(3,20)_ = 15.93, *p* < 0.0001, R^2^ = 0.7050). Differences in the p-mTOR s2448 and LC3B-II expression were also investigated, but they were shown to be non-significant (post hoc Dunnett’s, F _(3,20)_ = 0.09542, *p* < 1, R^2^ = 0.01411) and (post hoc Dunnett’s, F _(3,20)_ = 0.8772, *p* < 0.5, R^2^ = 0.1163) accordingly (Figure S1). Spearman’s rank correlations were then performed to investigate the possible relationship between T14 and autophagy markers. The T14 expression was positively correlated with the pTau (Ser202, Thr205) (Figure 2e, r = 0.8278, *p* < 0.0001), p62 (Figure 2f, r = 0.7057, *p* < 0.0003), and optineurin expression (Figure 2g, r = 0.5758, *p* < 0.004).

### 2.3. In Vitro Validation of Positive Autophagic Controls

**Figure 3:**
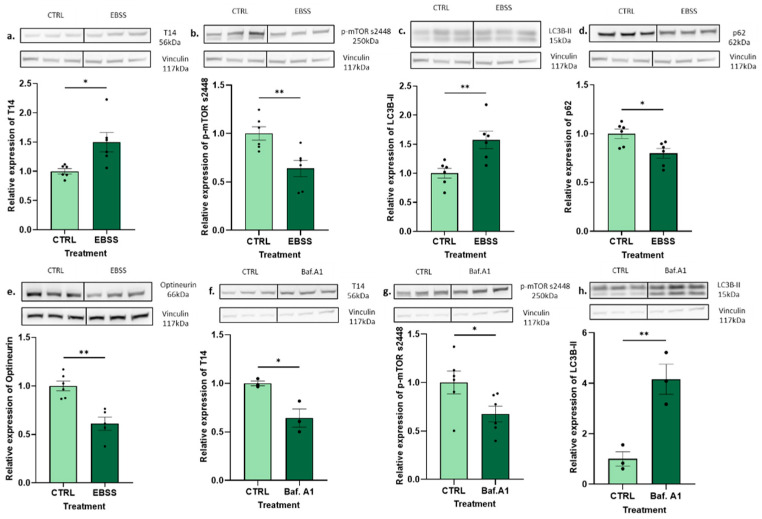
The effect of EBSS and Baf.A1 on T14 and autophagy markers. Protein expression after a 24 h EBSS (3 mL) treatment. (**a**) Expression of T14 (56 kDa) relative to vehicle control. (**b**) Expression of p-mTOR s2448 (250 kDa) relative to vehicle control. (**c**) Expression of LC3B-II (15 kDa) relative to vehicle control. (**d**) Expression of p62 (62 kDa) relative to vehicle control. (**e**) Expression of optineurin (66 kDa) relative to vehicle control. Protein expression after a 24 h Bafilomycin.A1 (100 nM) treatment. (**f**) Expression of T14 (56 kDa) relative to vehicle control. (**g**) Expression of p-mTOR s2448 (250 kDa) relative to vehicle control. (**h**) Expression of LC3B-II (15 kDa) relative to vehicle control. Representative Western blots and their corresponding vinculin (117 kDa) measurements are shown above each graph. All experiments were performed in PC12 cells, and all data were normalised to vinculin. The bars represent the mean, the errors represent the SEM, and the dots represent individual data points. Unpaired *t*-test, * *p* < 0.05, ** *p* < 0.01, *n* = 3–6 wells per treatment.

The correlation between T14 and autophagy in development and AD further supports the notion that they are linked, and that neurodegeneration may be an aberrant recapitulation of development. To prove the causality of the relationship, a more reductionist approach was used. In vitro studies were performed, including positive autophagic controls. These controls were used to demonstrate the efficiency of the autophagy markers but were independent of mTORC1. EBSS is a known stimulator of autophagy that acts by starving the cells [28]. The incubation of the PC12 cells with EBSS for 24 h significantly increased T14 compared to that in the vehicle-treated controls (Figure 3a, unpaired *t*-test, *p* < 0.02). The EBSS treatment also significantly decreased the mTORC1 expression (Figure 3b, unpaired *t*-test, *p* < 0.008) but increased LC3B-II (Figure 3c, unpaired *t*-test, *p* < 0.008), a marker of the autophagosome number. The autophagy markers p62 (Figure 3d, unpaired *t*-test, *p* < 0.02) and optineurin (Figure 3e, unpaired *t*-test, *p* < 0.02) were seen to significantly decrease after the treatment. On the other hand, Baf.A1 inhibits autophagic flux by blocking the fusion between lysosomes and autophagosomes, preventing lysosomal degradation [28]. The results from the PC12 cells show a decrease in T14 (Figure 3f, unpaired *t*-test, *p* < 0.03), followed by a decline in the mTORC1 expression (Figure 3g, unpaired *t*-test, *p* < 0.05). LC3B-II was seen to significantly increase (Figure 3h, unpaired *t*-test, *p* < 0.009); however, the aggregation of p62 (unpaired *t*-test, *p* < 0.8) and optineurin (unpaired *t*-test, *p* < 0.3) was shown to be non-significant (Figure S2). The cell viability of the treatments was measured, and no significant differences in the cell count were observed: EBSS (unpaired *t*-test, *p* < 0.7) and Baf.A1 (unpaired *t*-test, *p* < 0.09) (Figure S3).

### 2.4. T30 Promoted T14 and p-mTOR s2448 Expression and NBP14 Reversed the Effects

**Figure 4:**
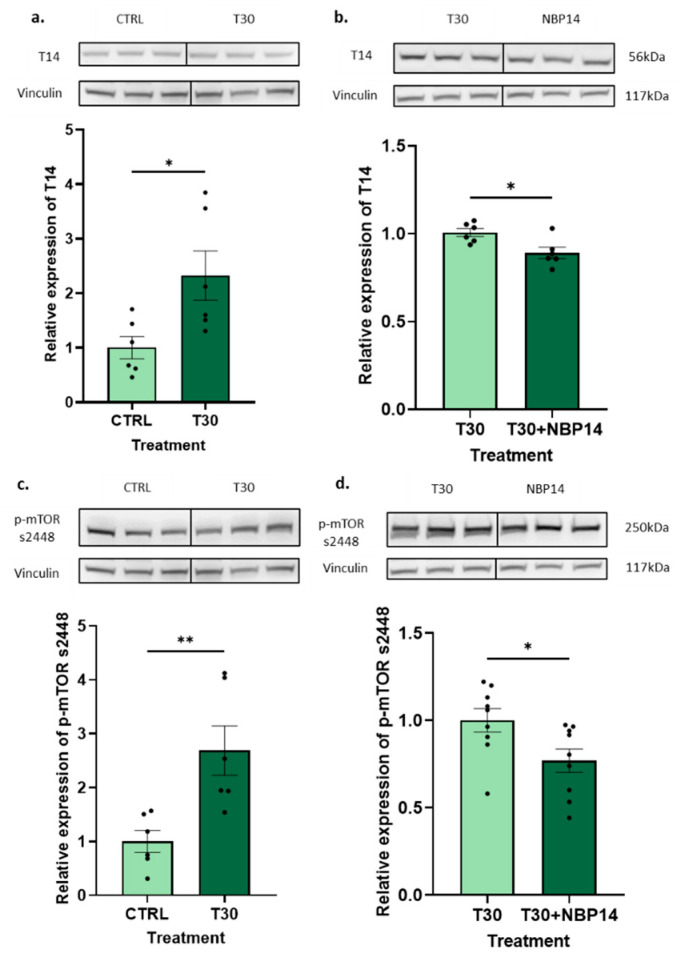
T30/T14 stimulate p-mTOR s2448 expression, which is blocked by NBP14. (**a**) Expression of T14 (56 kDa) after a 24 h T30 (100 nM) treatment, relative to vehicle control. (**b**) Expression of T14 (56 kDa) after a 24 h T30 (100 nM) + NBP14 (100 nM) treatment, relative to T30 (100 nM). (**c**) Expression of p-mTOR s2448 (250 kDa) after a 24 h T30 (100 nM) treatment, relative to vehicle control. (**d**) Expression of p-mTOR s2448 (250 kDa) after a 24 h T30 (100 nM) + NBP14 (100 nM) treatment, relative to T30 (100 nM). Representative Western blots for each protein and their corresponding vinculin (117 kDa) measurements are shown above each graph. All experiments were performed in PC12 cells, and all data were normalised to vinculin. The bars represent the mean, the errors represent the SEM, and the dots represent individual data points. Unpaired *t*-test, * *p* < 0.05, ** *p* < 0.01, *n* = 6–9 wells per treatment.

Initially, our previous work on T14 and mTORC1 was replicated to later investigate the association between T30 and NBP14 on autophagic flux via this mechanism. The treatment of the PC12 cells for 24 h with 100 nM of T30 showed a significant feedforward increase in T14 (Figure 4a, unpaired *t*-test, *p* < 0.03). We then wanted to confirm whether the administration of NBP14 would downregulate the T14 expression. The results showed a significant decrease in T14 after a 24 h T30 (100 nM) versus T30 (100 nM) + NBP14 (100 nM) treatment (Figure 4b, unpaired *t*-test, *p* < 0.02). Next, we measured the p-mTOR s2448 expression in the PC12 cell samples previously treated with 100 nM of T30. Here, the same increasing trend as in T14 was observed (Figure 4c, unpaired *t*-test, *p* < 0.008). NBP14 was seen to downregulate the p-mTOR s2448 expression (Figure 4d, unpaired *t*-test, *p* < 0.03). To validate the effects of T30, a treatment of 100 nM T15 was performed, having no effect on either the T14 (unpaired *t*-test, *p* < 0.6) or p-mTOR s2448 expression (unpaired *t*-test, *p* < 0.7) (Figure S4). The results were consistent with previous findings [10].

### 2.5. T30 Inhibits Autophagic Flux in PC12 Cells

**Figure 5:**
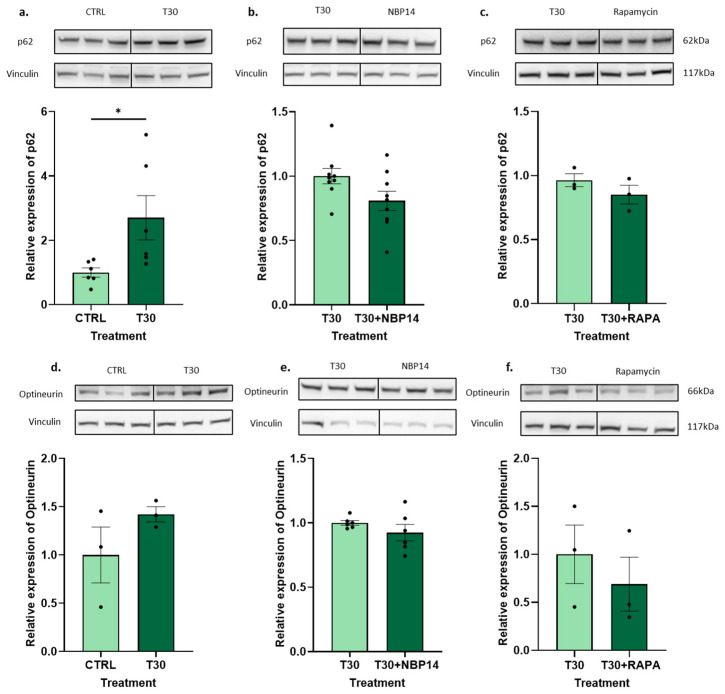
Effect of T30, NBP14, and rapamycin on autophagy markers. (**a**) Expression of p62 (62 kDa) after a 24 h T30 (100 nM) treatment, relative to vehicle control. (**b**) Expression of p62 (62 kDa) after a 24 h T30 (100 nM) + NBP14 (100 nM) treatment, relative to T30 (100 nM). (**c**) Expression of p62 (62 kDa) after a 24 h T30 (100 nM) + rapamycin (1 µM) treatment, relative to T30 (100 nM). (**d**) Expression of optineurin (66 kDa) after a 24 h T30 (100 nM) treatment, relative to vehicle control. (**e**) Expression of optineurin (66 kDa) after a 24 h T30 (100 nM) + NBP14 (100 nM) treatment, relative to T30 (100 nM). (**f**) Expression of optineurin (66 kDa) after a 24 h T30 (100 nM) + rapamycin (1 µM) treatment, relative to T30 (100 nM). Representative Western blots and their corresponding vinculin (117 kDa) measurements are shown above each graph. All experiments were performed in pheochromocytoma cell line 12 (PC12) cells, and all data were normalised to vinculin (117 kDa). The bars represent the mean, the errors represent the SEM, and the dots represent individual data points. Unpaired *t*-test, * *p* < 0.05, *n* = 3–9 wells per treatment.

The known correlation between the T14 and p-mTOR s2448 expression [10] and the role of mTORC1 in autophagy [1,4,29,30,31] led us to perform the following experiments. Here, we measured the effects of 100 nM of T30 on two different autophagy markers: p62 and optineurin. The Western blot analysis showed a significant increase in p62 (Figure 5a, unpaired *t*-test, *p* < 0.04) after a 24 h treatment. The p62 expression after a T30 (100 nM) versus T30 (100 nM) + NBP14 (100 nM) treatment showed evidence of a partial reversal of the effects; however, the decrease was not significant (Figure 5b, unpaired *t*-test, *p* < 0.07). A non-significant change in the same direction was observed after a 1 µm rapamycin treatment (Figure 5c, unpaired *t*-test, *p* < 0.3). The optineurin measurements showed the same tendency as p62 after the T30 (Figure 5d, unpaired *t*-test, *p* < 0.3), NBP14 (Figure 5e, unpaired *t*-test, *p* < 0.3), and rapamycin (Figure 5f, unpaired *t*-test, *p* < 0.5) treatments. However, the increase in T30 against the vehicle control group was not significant. LC3B-II was also measured to identify any significant changes in the autophagosome numbers after the treatments. The vehicle control versus T30 treatment showed a significant increase in LC3B-II (unpaired *t*-test, *p* < 0.04). NBP14 and rapamycin then restored the baseline values (unpaired *t*-test, *p* < 0.4) (unpaired *t*-test, *p* < 0.5) accordingly (Figure S5). To corroborate the significance of these results, a treatment of 100 nM T15 was performed, having no effects on the LC3B-II (unpaired *t*-test, *p* < 1), p62 (unpaired *t*-test, *p* < 0.7), or optineurin expression (unpaired *t*-test, *p* < 0.7) (Figure S4). Additionally, the cell viability of all the treatments was measured, and no significant differences in the cell count were observed: T30 (unpaired *t*-test, *p* < 0.7), NBP14 (unpaired *t*-test, *p* < 0.3), rapamycin (unpaired *t*-test, *p* < 0.06), and T15 (unpaired *t*-test, *p* < 0.7) (Figure S3).

## 3. Discussion

Here, we show that the overexpression of T14 through in vitro treatments causes an increase in p-mTOR s2448, pTau (Ser202, Thr205), and autophagy marker expression. Even though the present study has established that the same tendency is observed in postmortem AD tissue and that there is a decline in the autophagosome number across Braak stages, an aggregation of polyubiquinated substances within those autophagosomes has not been identified. We also confirmed a correlation between T14 and phosphorylated Tau at residues Ser202 and Thr205, as well as p62 and optineurin expression in developing mouse samples. This implies that as the T14 levels naturally decrease in early development, the autophagic mechanism is enhanced, further confirming the notion that T14 hinders the normal functioning of this mechanism via the aggregation of ubiquitin-positive inclusion proteins inside autophagosomes. Finally, we demonstrate that the administration of NBP14 in vitro reverses the effects of T30, selectively blocking mTORC1 and enhancing the natural autophagic flux to the same degree as that of rapamycin. 

### 3.1. Methodological Considerations

Western blotting was considered appropriate for the measurement of T14, pTau, mTORC1, and autophagy markers in the brain. The proteins of interest were identified at different band sizes; for this reason, the same membrane was stained with multiple antibodies, eliminating cross-experimental inconsistencies. However, the exclusive reliance on protein biochemistry was a primary constraint in this study. Although this methodology is widely accepted for relative protein quantification [32], fully quantitative methods like enzyme-linked immunosorbent assay (ELISA) could be employed to determine the absolute protein concentrations across different tissues and cell lines.

On the other hand, the T14 antibody is polyclonal and specific to its full sequence, not recognising it when the C-terminal lysine is shortened by tryptophan or is absent; it also does not detect AChE or T30 expression [5]. The specificity of the T14 antibody has been previously established by complexion with exogeneous T14 via immunoneutralization, where it eradicated all immunohistochemistry staining [33]. Hippocampal samples from AD patients were used to determine whether the increased expression of T14 and p-mTOR s2448 across Braak stages [26] correlated with a decline in autophagic flux. It has been suggested that the aberrant activation of T14 stimulates cell loss at the pre-symptomatic stages of the disease; this is initiated in the isodendritic core (IC) [14]. The pathology is then spread across other brain regions, such as the hippocampus, which presents tau aggregation from stage II [34]. T14 has been shown to be bioactive in rat hippocampal organotypic cultures [8], as well as in the hippocampus of 5XFAD transgenic mice [33], stimulating neuronal degeneration. Additionally, T14 measurements in the hippocampal region of postmortem AD brains were seen to significantly increase from the early (Braak 0-II) to late (Braak V-VI) stages [33]. As previously shown, T14 can alter the mTORC1 expression [10], and this correlation was maintained in the hippocampus of AD patients, where there was also an increase in p-mTOR expression [35]. The initiation of this pathway has been associated with an impairment in autophagy, commonly observed in ageing and neurodegeneration [36,37]. The induction of autophagy in hippocampal neurons via injections of pharmacological and genetic modulators of the mechanism enhances memory formation and activity-dependent synaptic plasticity, reversing age-impaired memory [37]. Therefore, the hippocampus was selected as the region of interest to evaluate the effect of T14 on autophagic flux.

Meanwhile, brain tissue from developing mice was used to explore the possible link between T14 activity and autophagic flux. Mice are commonly used as model organisms in the study of molecular genetics of mammalian development. The mouse genome is the same size as the one found in humans, and there is virtually a one-to-one resemblance between their genes [38]. Human proteins have been found to be 80–90% identical in their amino acid sequences; in addition, larger nucleotides have shown an evident similarity via the comparison of DNA sequences [38]. Moreover, mouse brain tissue effectively mimics the elevated T14 levels found during early development. Therefore, the use of this rodent was deemed to be appropriate [39].

Finally, PC12 cells were used in this study to trace the intracellular steps linking autophagy and the T14 signalling pathway. These cells are derived from a pheochromocytoma cell line originating from the neural crest of the rat adrenal medulla [40,41]. They are known to release multiple neurotransmitters [42], which are useful in the study of neuronal function and neurodegeneration in vitro [40,42,43]. Additionally, the adrenal medulla of AD patients displayed similar pathological features to the ones observed in the brain: neurofibrillary tangles (NFTs) and paired helical filaments [44]. Exogenous T30/T14 selectively shifted a sub-threshold dose of Aβ to a toxic effect, which also developed in the comparable release of acetylcholinesterase in PC12 cells [19]. Here, we stimulated T14 expression to activate mTORC1 activity and, in turn, inhibit autophagic flux in PC12 cells.

### 3.2. Association Between the Autophagic Mechanisms and the T14 Signalling Pathway

Autophagy is a physiological process by which the lysosome degrades cytoplasmic components, such as organelles and macromolecules, while others are recycled for cellular repair [29] (see graphical abstract). Increasing evidence suggests that it also has preserved functions in differentiation and development [45]. Moreover, this process plays a key role in survival during neonatal starvation in erythropoiesis, lymphopoeisis, and adipogenesis, as well as a homeostatic function in terminally differentiated cells like neurons [45]. The knockout of autophagy-related (*Atg*) genes (e.g., *Atg5* or *Atg7*) revealed gradual neuronal degradation and the aggregation of abnormal cellular proteins and ubiquitin-positive inclusion bodies (e.g., p62), as well as Aβ and pTau in neurons [31,46]. The subsequent blockade of the autophagic process stimulates tissue degeneration and the build-up of Aβ autophagic vacuoles [47], demonstrating once again its fundamental function in post-mitotic differentiated cells [31]. Electron microscopy identified the accumulation of immature autophagosomes and dystrophic neurites in AD brains prior to Aβ plaque development [30]. Furthermore, these clusters were observed in neuronal dendrites, indicating defective axonal transportation [30]. Another study suggested that p62 accumulation could be one of the main drivers of cellular toxicity in the liver and have a similar effect on the brain [48]. Additionally, p62 overexpression causes the abnormal hyperactivation of nuclear factor erythroid 2-related factor 2 (Nrf2) [49]. Consequently, the excessive secretion of reactive oxygen species (ROS) and clusters of dysfunctional mitochondria damage ROS-sensitive cells like dopaminergic neurons, leading to neurodegeneration [50]. Mitochondrial dysfunction and ROS release are also known products of mTORC1 [13], which is stimulated by GSK-3 [10,11] after the calcium influx caused by T14 (see graphical abstract) [8,17,18,19]. In the later stages of the disease, the mTORC1 and T14 expression in the brain increase, suggesting that their presence may be connected [10].

The mTORC1 pathway and T14/T30 have been associated with AD and cancer stimulation [13,22,51]; hence, it was hypothesised that the hyperactivation of the mechanism in both pathologies initiated from the same unidentified trigger [52]. T30 enhances neurite growth through protein and autophagosome synthesis [53] via mTORC1 activation. In contrast to these anabolic processes, mTORC1 hinders the catabolic stages of autophagy through the phosphorylation-dependent inhibition of ULK1 and *Atg13* [6,7,41]. In turn, this enhanced mTORC1 activity will inhibit the transcription factor EB (TFEB) family members, limiting its degradative capacity [54], contributing to mTORopathies linked to neuroplasticity [55]. Previous literature has also observed alterations in TFEB levels in a Braak-stage-related manner and a decrease in these in the later stages of the disease [56]. Parkinson’s disease models have found the blockade of the mTOR pathway to be beneficial, as α-synuclein aggregates were not recognised as a major trigger of the pathology [57].

Additionally, multiple studies that have used AD models and Alzheimer-like pathology have emphasised the effectiveness of rapamycin in autophagy induction via aggregate clearance [57,58,59,60,61]. Given the correlation between T14 and p-mTOR in human midbrain samples from AD patients [10], as well as their involvement in the inhibition of autophagy and neurodegeneration, the cyclic variant NBP14 could potentially be used to reverse these effects. Where it is predicted to have superior therapeutic potential to rapamycin in the blockade of p-mTOR [10]. More generally, the therapeutic potential of NBP14 has been validated in vitro [7], in vivo [33], and ex vivo [62].

## 4. Materials and Methods

### 4.1. PC12 Cell Culture

Firstly, 100 mm dishes (StarLab) were coated with type IV collagen from human placenta (Sigma Merck, Burlington, MA, USA) to plate wild-type PC12 cells (Sigma Merck). PC12 cells are an immortalised pheochromocytoma cell line originating from the rat adrenal medulla [40]. These are commonly used due to their ability to display sympathetic neuron behaviour when exposed to nerve growth factors (NGFs) [43]. As previously described [10], the dishes were cultured in full growth medium with high-glucose Dulbecco’s Modified Eagle’s Medium (DMEM) (Thermofisher, Waltham, MA, USA) and supplemented with 2.5 μg/mL Amphotericin B, 1:100 Penicillin/Streptomycin, 5% Foetal Bovine Serum (FBS), and 10% heat-inactivated Horse Serum (HS). Cells were cultured in a humidified incubator at 37 °C with 5% CO_2_, and the medium was replaced every 2 days. During the passage, cells were scraped from the 100 mm dish via a cell scraper and then passed through a needle and a syringe twice, with a portion of them being kept for plating onto a new dish. The passage number ranged between 14 and 30. Treatments with the following substances were performed: Earl’s Balanced Salt Solution (EBSS), synthesised by Sigma Merk (Darmstadt, Germany, E2888); Bafilomycin.A1 (Baf.A1), synthesised by Abcam (Cambridge, UK, 88899-55-2); and rapamycin, synthesised by Bio-Techne (Minneapolis, MN, USA, 1292). Both peptides were reconstituted in dimethyl sulfoxide (DMSO). T30 was synthesised by Genosphere Biotechnologies (Boulogne-Billancourt, France) and NBP14 by Bachem (Bubendorf, Switzerland); they were then reconstituted in acetonitrile (Act) and distilled water (dH_2_0) accordingly. All treatments were performed using a concentration of 100 nM, except rapamycin, which was used at 1 µM.

### 4.2. Animals

Twenty-four male and female juvenile C57BL/6J mice (Charles River) were purchased at postnatal day 7 (P7), P14, P21, and P60 (*n* = 6/age). Immediately upon arrival at the holding facility, mice were deeply anaesthetised with an overdose of isoflurane and killed by Schedule 1 cervical dislocation and exsanguination. Experiments followed the principles of the Animal Research: Reporting of In Vivo Experiments (ARRIVE) and were performed according to the United Kingdom Animals (Scientific Procedures) Act of 1986. Brains were snap frozen in dry-ice cooled isopentane and stored at −80 °C.

### 4.3. Human Clinical Samples

For human tissue experiments, an ethics application was approved by London—City and East NRES Committee on behalf of the Human Tissue Bank of Oxford Radcliffe Hospital NHS that complied with the Human Tissue Act, Human Tissue Authority Codes of Practice, and other laws relevant to postmortem examinations and use of tissue. Ethics committee number: 08/H0704/128+5. Approval code: 07/Q1605/16. Approval date: 2 December 2015.

The first cohort comprised 21 patients, aged 80 to 95 (*n* = 4/Braak stage). Power analysis was performed on the first group of samples to select a second cohort. A total of 62 hippocampal region samples across three Braak stages—control (Braak 0/I/II), Braak III/IV, and Braak V/VI—were selected with age constraints between 85 and 90 years old. Both cohorts were combined to acquire a total of 83 hippocampus samples.

### 4.4. Protein Extraction and Quantification

Human brain samples were initially weighed, and the mass was recorded. A volume of 1 mL of the same PBS lysis buffer was added per 300 mg of tissue. If samples were ≤200 mg, then an additional 500 µL of tissue buffer was added. Loose and tight-fit pestles were used to homogenise the tissue, and this was centrifuged for 40 min at 13,000 rpm at 4 °C. Mouse brains were either taken whole (P7) or sliced down the longitudinal fissure (P14, P21, and P60). The right hemispheres were homogenised with 500 μL of lysis buffer containing PBS (1× Solution, pH 7.4, Fisher BioReagents™) supplemented with phosphatase inhibitor cocktail (PhosSTOP, Roche, Basel, Switzerland) and protease inhibitor cocktail (cOmplete™ ULTRA Tablets, Mini, EDTA-free, EASYpack Protease Inhibitor Cocktail, Roche) per 100 mg of tissue. Samples were then sonicated for 5 min and centrifuged for 30 min at 13,000 rpm at 4 °C.

After their corresponding treatments, PC12 cells were harvested with 1 mL phosphate-buffer saline (PBS) (Thermofisher). They were then detached from the plate using a cell scraper. Samples were centrifuged at 500× *g* for 5 min at 4 °C and then lysed with radio-immunoprecipitation assay (RIPA) buffer (1× Solution, pH 7.8–8.2, Sigma Merk). They were centrifuged again for 10 min at 13,000 rpm at 4 °C. Protein concentration of all samples was calculated using a Pierce™ Bovine Serum Albumin Standard Set (Thermofisher) and Pierce™ 660 nm Protein Assay Reagent (Fisher Scientific, Pittsburgh, Pennsylvania, PA, USA) [5]. Absorbance was assessed at 660 nm using a CLARIOstar Plus plate reader (BMG Labtech, Aylesbury, UK) and analysed in GraphPad Prism (GraphPad software 9.4.1, San Diego, CA, USA).

### 4.5. Bis-Tris Western Blot

Samples were prepared for Bis-Tris Western blot analysis through the addition of equal concentrations of protein (15 μg for PC12 cells and mouse samples, and 25 μg for human hippocampus) to 10× Invitrogen Bolt Sample Reducing Agent (Fisher Scientific) plus 4× Invitrogen Bolt LDS Sample Buffer and heated for 10 min at 60 °C. They were then loaded for electrophoresis into 4–12% Bis-Tris Plus Mini Protein Gels with 20× Bolt MES SDS Running Buffer. Invitrogen SeeBlue Plus2 Pre-Stained Standard ladder was used for protein band size identification (all reagents obtained from ThermoFisher). The gels were then transferred onto a Polyvinylidene difluoride (PVDF) membrane (0.2 μM pore size; Bio-rad) using 20× Invitrogen Bolt Transfer Buffer (ThermoFisher). Membranes were briefly stained for 5 min with Ponceau S Solution (Fisher Scientific) to determine the total protein and imaged with a CCD camera (G-Box, Syngene, Cambridge, UK). The membranes were then blocked for 1 h at room temperature with 5% milk solution in tris-buffered saline (TBS) with 0.05% TWEEN20 (Sigma Merk) (TBS-T). Afterwards, they were incubated in 5% milk solution with the following primary antibodies: rabbit anti-T14 (Genosphere Biotechnologies, Boulogne-Billancourt, France, 1:1000) [5], rabbit anti-mTORC1 p-mTOR S2448 (Abcam, Cambridge, UK, ab109268, 1:5000), rabbit anti-light chain 3B (LC3B) polyclonal (Thermofisher, PA1-46286, 1:5000), rabbit anti-p62/SQSTM1 polyclonal (Thermofisher, PA5-20839, 1:5000), rabbit anti-optineurin polyclonal (Thermofisher, 711879, 1:5000), mouse anti-phospho-Tau AT8 (Ser202, Thr205; pTau) (ThermoFisher, MN1020, 1:1000), and rabbit anti-vinculin (Abcam, EPR8185, 1:10,000). They were then left overnight at 4 °C with gentle agitation.

Membranes were washed with TBS-T three times for 5 min and incubated in 5% milk solution with goat anti-rabbit IgG (H+L) secondary antibody (ThermoFisher, G-21234) or goat anti-mouse (H+L) cross-absorbed secondary antibody, HRP (ThermoFisher, 32230) at 1:10,000 for 1 h at room temperature with gentle agitation. Membranes were then washed in TBS-T three times for 5 min and once with TBS for 5 min. They were then imaged using the Clarity Western ECL Substrate (Bio-rad, Berkley, California, CA, USA) with the CCD Camera (G-Box, Syngene, Cambridge, UK). Membranes stained with anti-mTORC1 p-mTOR S2448, anti-vinculin, and anti-LC3B were imaged for 20 s. Anti-phosphoTau AT8 (Ser202, Thr205), anti-p62/SQSTM1, and anti-optineurin stains were imaged for 2 min. Anti-T14 stains required a 40 min exposure. Membranes included lanes only stained with secondary antibodies to control for non-specific binding, and they were cut to stain for both the protein of interest and the loading control for each sample.

### 4.6. Western Blot Analysis

Band intensities were quantified using ImageJ (NIH) Version 1.54k. Human hippocampus samples were normalised to total protein (ponceau) stain. Data points were normalised to the Control/Braak stage I/II (CTRL/BI/BII) cohort to account for variation between gels. Mouse and PC12 cell samples were normalised to vinculin as a loading control. Due to variation in the absolute signal intensity levels between membranes, mouse data were also normalised to the mean P7 value and PC12 cell samples to the average control treatment per membrane.

### 4.7. Statistical Analysis

The normality of data was assessed with D’Agostino Pearson’s test; data were parametric, so unpaired *t*-test and one-way ANOVA with Tukey’s or Dunnett’s post hoc test were used. Outliers were identified and removed using ROUT analysis. Spearman’s rank correlations were used to correlate T14 with the expression of other proteins. GraphPad Prism (GraphPad software 9.4.1, San Diego, CA, USA) was used for statistical analysis, and *p* < 0.05 is considered statistically significant throughout.

## 5. Conclusions

This study shows that T14 inhibits autophagic flux via the selective activation of the mTORC1 pathway and the downstream intracellular events associated with the mechanism, further demonstrating that the aberrant reactivation of the peptide stimulates the development of pathologies like AD. This correlation has been shown in vitro, as well as in growth and neurodegeneration. We suggest that the displacement of T14 in α-7 nAChR via its cyclic variant NBP14 and the simultaneous blockade of the mTORC1 pathway present a partial reversal of the effects on autophagy. Further experiments would benefit from the use of transgenic mouse models such as 5XFAD. Here, autophagic flux between wild-type, 5XFAD-vehicle, and 5XFAD-NBP14 mice could be investigated at an mRNA and protein level. Overall, these experiments would further support the notion that T14 hinders the autophagic mechanism and NBP14 enhances it.

## Data Availability

All the data supporting the findings of this study are available within the paper.

## Supplementary Materials

The following supporting information can be downloaded at: https://www.mdpi.com/article/10.3390/ijms252312837/s1.
