# Supplementary material for: A Novel 14mer Peptide Inhibits Autophagic Flux via Selective Activation of the mTORC1 Signalling Pathway: Implications for Alzheimer’s Disease
Source: Int J Mol Sci. 2024 Nov 29;25(23):12837. doi: 10.3390/ijms252312837 (PMC11641777; doi:10.3390/ijms252312837)
Supplement: Supplementary file 1 [file ijms-25-12837-s001.zip › ijms-3308714-supplementary.pdf]

Supplementary Figure S1

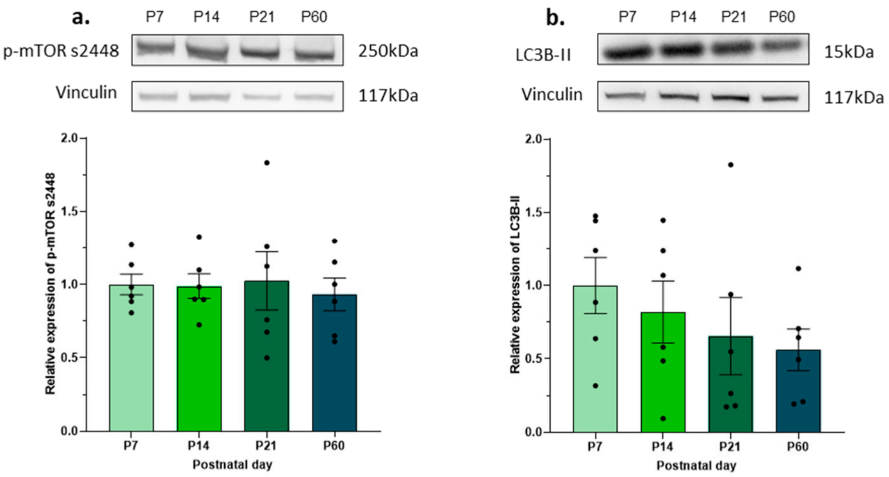

Figure S1. Measurement of p-mTOR s2448 and LC3B-II in developing murine brain. (**a**) Expression of p-mTOR s2448 (250 kDa) during development relative to the mean of P7. (**b**) Expression of microtubule-associated protein 1 light chain 3 beta phosphatidylethanolamine (LC3B-II) (15 kDa) during development relative to the mean of P7. Representative Western blots for each protein and their corresponding vinculin (117 kDa) measurements are shown above each graph. All data were normalised to vinculin. The bars represent the mean, the errors represent the SEM, and the dots represent the individual data points of P7, P14, P21, and P60 mice. One-way ANOVA with post hoc Dunnett's tests, *$p < 0.05$, $n = 6$/age.

Supplementary Figure S2

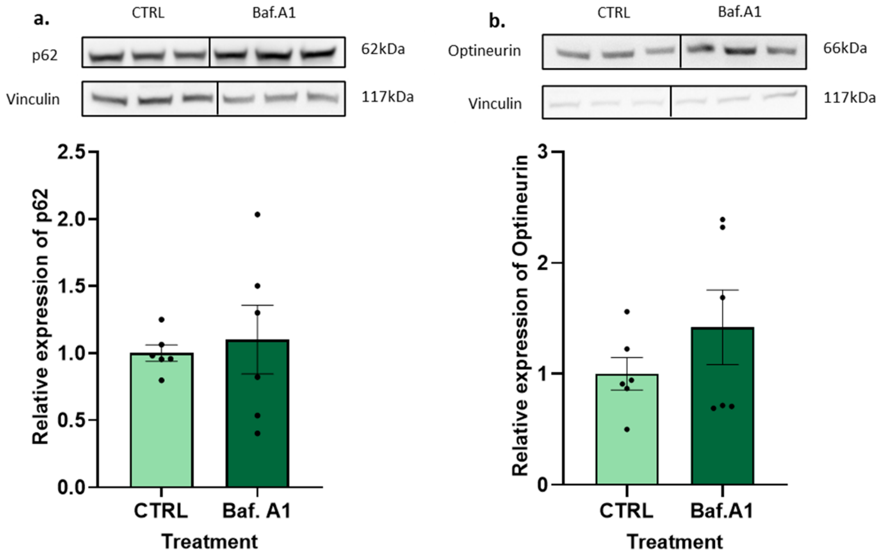

Figure S2. The effects of Baf.A1 on p62 and optineurin. (**a**) Expression of p62 (62 kDa) after a 24 h Baf. A1 (100 nM) treatment. (**b**) Expression of optineurin (66 kDa) after a 24 h Baf. A1 (100 nM) treatment. Expression of all proteins was relative to vehicle control. Representative Western blots and their corresponding vinculin (117 kDa) measurements are shown above each graph. All experiments were performed in PC12 cells, and all data were normalised to vinculin. The bars represent the mean, the errors represent the SEM, and the dots represent individual data points. Unpaired *t*-tests were performed, * $p < 0.05$, and $n = 6$ wells per treatment.

Supplementary Figure S3

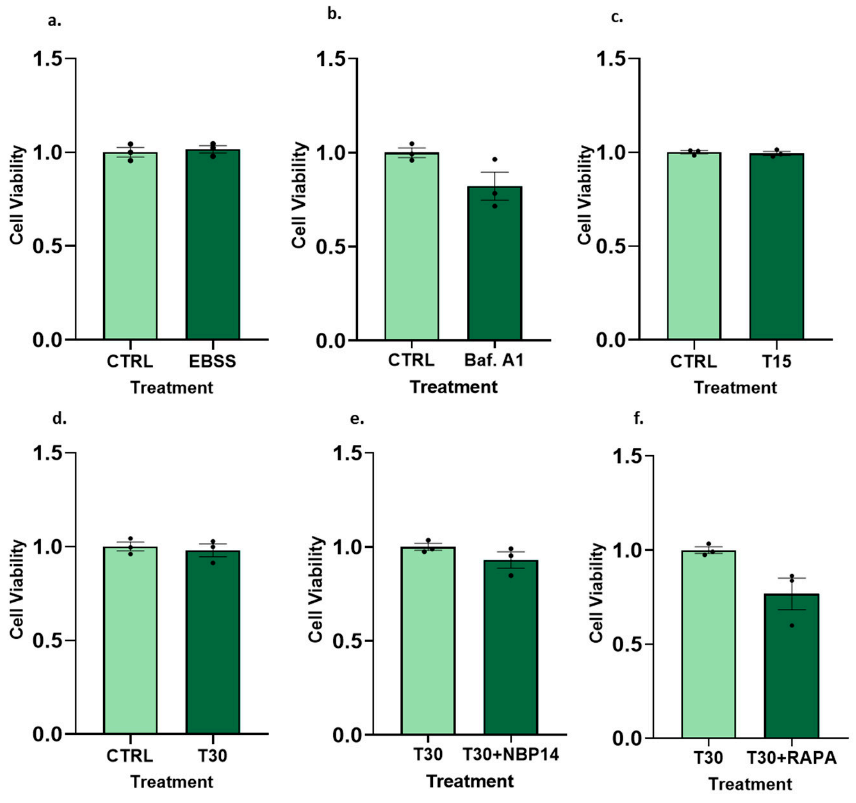

Figure S3. Cell viability across PC12 treatments presents no alterations. (**a**) Cell viability after a 24 h EBSS (3 ml) treatment, relative to vehicle control. (**b**) Cell viability after a 24 h Baf. A1 (100 nM) treatment, relative to vehicle control. (**c**) Cell viability after a 24 h T15 (100 nM) treatment, relative to vehicle control. (**d**) Cell viability after a 24 h T30 (100 nM) treatment, relative to vehicle control. (**e**) Cell viability after a 24 h T30 (100 nM) + NBP14 (100 nM), relative to T30 (100 nM). (**f**) Cell viability after a 24 h T30 (100 nM) + rapamycin (1 μM), relative to T30 (100 nM). Cells were manually counted and presented as the ratio of alive cells per total count. The bars represent the mean, the errors represent the SEM, and the dots represent individual data points. Unpaired *t*-tests were performed, * $p < 0.05$, and $n = 3$ wells per treatment.

Supplementary Figure S4

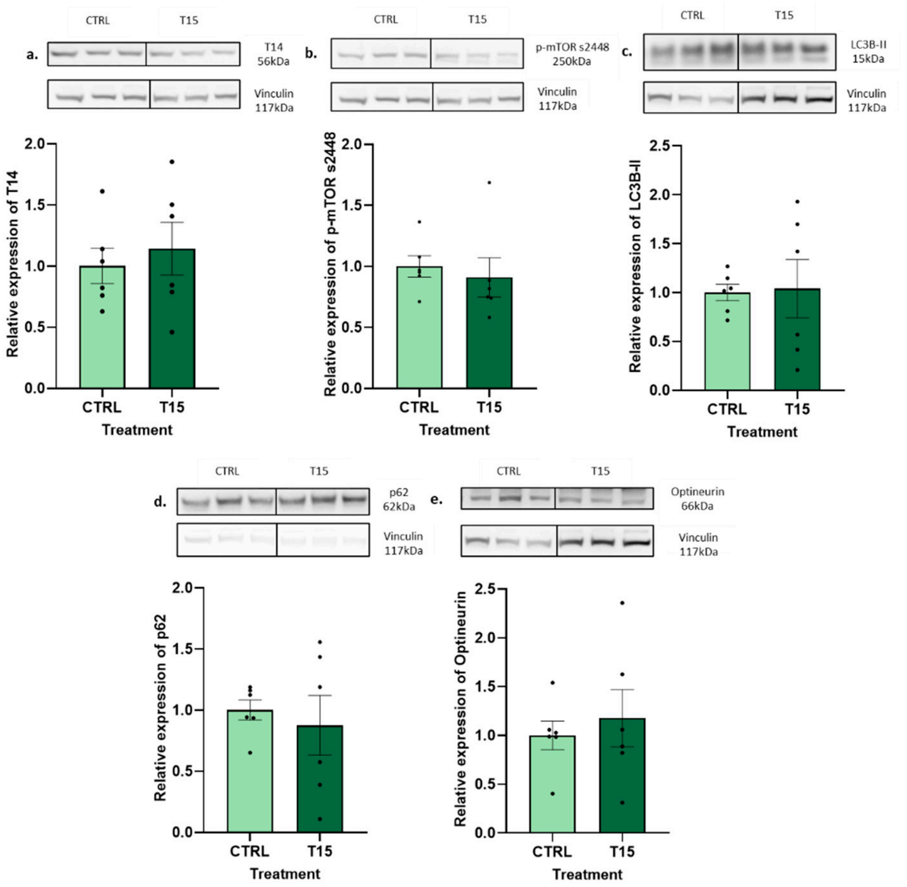

Figure S4. T15 treatments present no alterations in protein expression. (**a**) Expression of T14 (56 kDa) after a 24 h T15 (100 nM) treatment. (**b**) Expression of p-mTOR s2448 (250 kDa) after a 24 h T15 (100 nM) treatment. (**c**) Expression of LC3B-II (15 kDa) after a 24 h T15 (100 nM) treatment. (**d**) Expression of p62 (62 kDa) after a 24 h T15 (100 nM) treatment. (**e**) Expression of optineurin (66 kDa) after a 24 h T15 (100 nM) treatment. Expression of all proteins was relative to vehicle control. Representative Western blots and their corresponding vinculin (117 kDa) measurements are shown above each graph. All experiments were performed in PC12 cells, and all data were normalised to vinculin (117 kDa). The bars represent the mean, the errors represent the SEM, and the dots represent individual data points. Unpaired *t*-tests were performed, * $p < 0.05$, and $n = 6$ wells per treatment.

Supplementary Figure S5

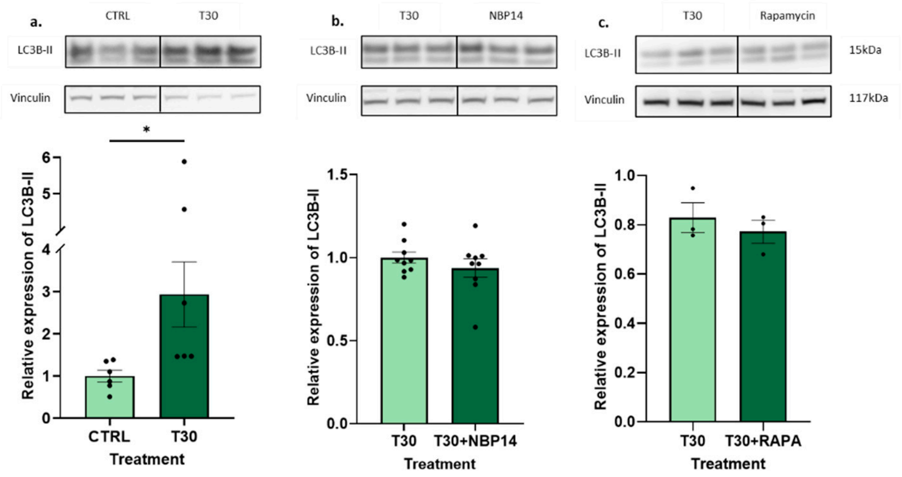

Figure S5. T30 causes autophagy marker aggregation, and NBP14 and rapamycin attenuate this increase. (**a**) Expression of LC3B-II (15 kDa) after a 24 h T30 (100 nM) treatment, relative to vehicle control. (**b**) Expression of LC3B-II (15 kDa) after a 24 h T30 (100 nM) + NBP14 (100 nM) treatment, relative to T30 (100 nM). (**c**) Expression of LC3B-II (15 kDa) after a 24 h T30 (100 nM) + rapamycin (1 μM) treatment, relative to T30 (100 nM). Representative Western blots and their corresponding vinculin (117 kDa) measurements are shown above each graph. All experiments were performed in PC12 cells, and all data were normalised to vinculin (117 kDa). The bars represent the mean, the errors represent the SEM, and the dots represent individual data points. Unpaired *t*-test, * $p < 0.05$, n = 3-9 wells per treatment.
